# Supplementary material for: Endangered Exotic Pets on Social Media in the Middle East: Presence and Impact
Source: Animals (Basel). 2019 Jul 24;9(8):480. doi: 10.3390/ani9080480 (PMC6720740; doi:10.3390/ani9080480)
Supplement: Supplementary file 1 [file animals-09-00480-s001.pdf]

Table S1. Complete list of emojis included in comment compilation from celebrity social media posts based in the Middle East featuring assessed exotic animal species (Arabian oryx, giraffe, falcon, elephant, cheetah, orangutan, chimpanzee, tiger, and zebra).

Description	n	Expansive category	Condensed category
red heart	1847	love	positive
face with tears of joy	1664	happy	positive
smiling face with heart-eyes	1488	love	positive
two hearts	947	love	positive
rose	538	miscellaneous	neutral
face blowing a kiss	445	love	positive
thumbs up	445	happy	positive
clapping hands	417	happy	positive
sparkling heart	337	love	positive
revolving hearts	302	love	positive
smiling face with smiling eyes	296	happy	positive
tulip	294	miscellaneous	neutral
folded hands	289	miscellaneous	neutral
blue heart	274	love	positive
heart suit	264	love	positive
smiling face	231	happy	positive
ok hand	226	happy	positive
hugging face	214	love	positive
see-no-evil monkey	195	shock	neutral
growing heart	191	love	positive
kiss mark	181	love	positive
rolling on the floor laughing	177	happy	positive
winking face	150	happy	positive
loudly crying face	133	happy	positive
crown	128	miscellaneous	neutral
mouth	121	love	positive
smiling face with open mouth &amp- cold sweat	117	shock	neutral
grinning face with smiling eyes	110	happy	positive
cherry blossom	108	miscellaneous	neutral
smiling face with open mouth &amp- smiling eyes	104	happy	positive
smiling face with halo	101	happy	positive
hot beverage	100	miscellaneous	neutral
yellow heart	99	love	positive
dove	94	animal	neutral

broken heart	92	sad	negative
kissing face with smiling eyes	92	love	positive
balloon	91	miscellaneous	neutral
hibiscus	91	miscellaneous	neutral
beating heart	90	love	positive
heart decoration	85	love	positive
kissing face with closed eyes	83	love	positive
unamused face	83	anger	negative
flexed biceps	79	miscellaneous	neutral
wilted flower	73	miscellaneous	negative
lion face	72	animal	neutral
purple heart	71	love	positive
sparkles	71	miscellaneous	neutral
face with stuck-out tongue &amp- winking eye	68	happy	positive
heart with ribbon	67	love	positive
thought balloon	66	miscellaneous	neutral
victory hand	65	miscellaneous	neutral
face screaming in fear	64	anger	negative
leaf fluttering in wind	63	miscellaneous	neutral
grinning face	62	happy	positive
musical notes	62	miscellaneous	neutral
green heart	61	love	positive
pensive face	61	anger	negative
speak-no-evil monkey	61	miscellaneous	negative
smiling face with open mouth &amp- closed eyes	56	happy	positive
sleeping face	55	miscellaneous	negative
united arab emirates	55	geographic region	neutral
face savouring delicious food	54	happy	positive
female sign	52	miscellaneous	neutral
flushed face	52	happy	positive
raising hands	52	miscellaneous	neutral
sun	51	miscellaneous	neutral
couple with heart	50	love	positive
glowing star	50	miscellaneous	neutral
deciduous tree	49	miscellaneous	neutral
face with open mouth &amp- cold sweat	49	anger	negative
waving hand	47	miscellaneous	neutral
sun with face	45	miscellaneous	neutral

cloud	44	miscellaneous	neutral
eyes	44	miscellaneous	neutral
smiling cat face with heart-eyes	43	love	positive
bouquet	41	miscellaneous	neutral
crying cat face	40	sad	negative
smiling face with open mouth	40	happy	positive
crying face	39	sad	negative
maple leaf	39	miscellaneous	neutral
relieved face	39	happy	positive
hushed face	36	shock	neutral
face with stuck-out tongue	34	happy	positive
thinking face	34	anger	negative
backhand index pointing right	33	miscellaneous	neutral
eagle	32	animal	neutral
elephant	32	animal	neutral
face with rolling eyes	32	anger	negative
cat face with tears of joy	31	happy	positive
love letter	31	miscellaneous	neutral
musical score	31	miscellaneous	neutral
party popper	31	miscellaneous	neutral
blossom	30	miscellaneous	neutral
heart with arrow	30	love	positive
milky way	30	miscellaneous	neutral
horse	29	animal	neutral
person biking	29	miscellaneous	neutral
slightly smiling face	29	happy	positive
camel	26	animal	neutral
disappointed face	26	sad	negative
girl	25	miscellaneous	neutral
person getting massage	25	miscellaneous	neutral
weary face	25	anger	negative
male sign	24	miscellaneous	neutral
white medium star	23	miscellaneous	neutral
boy	22	miscellaneous	neutral
camera	22	miscellaneous	neutral
christmas tree	22	miscellaneous	neutral
person running	22	miscellaneous	neutral
black heart	21	love	negative
confused face	21	sad	negative
evergreen tree	21	miscellaneous	neutral
grimacing face	21	anger	negative

musical note	21	miscellaneous	neutral
oncoming fist	21	miscellaneous	negative
face with open mouth	20	shock	neutral
fire	20	miscellaneous	neutral
man	20	miscellaneous	neutral
person gesturing ok	20	miscellaneous	positive
person shrugging	20	miscellaneous	negative
red circle	20	miscellaneous	negative
wind face	20	miscellaneous	neutral
zzz	20	miscellaneous	negative
first quarter moon with face	19	miscellaneous	neutral
person wearing turban	19	miscellaneous	neutral
sparkler	19	miscellaneous	neutral
sunflower	19	miscellaneous	neutral
trophy	19	miscellaneous	neutral
anguished face	18	anger	negative
fearful face	18	sad	negative
monkey	18	animal	neutral
rainbow	18	miscellaneous	neutral
blond-haired person	17	miscellaneous	neutral
ceuta &amp- melilla	17	geographic region	neutral
four leaf clover	17	miscellaneous	neutral
kiss	17	love	positive
kissing face	17	love	positive
top arrow	17	miscellaneous	neutral
water wave	17	miscellaneous	neutral
astonished face	16	shock	negative
azerbaijan	16	geographic region	neutral
backhand index pointing up	16	miscellaneous	neutral
crescent moon	16	miscellaneous	neutral
face with stuck-out tongue &amp- closed eyes	16	happy	positive
globe showing asia-australia	16	miscellaneous	neutral
heavy heart exclamation	16	love	positive
megaphone	16	miscellaneous	neutral
person facepalming	16	anger	negative
smiling face with sunglasses	16	happy	positive
confounded face	15	anger	negative
ghost	15	miscellaneous	neutral

herb	15	miscellaneous	neutral
honey pot	15	miscellaneous	neutral
night with stars	15	miscellaneous	neutral
open hands	15	miscellaneous	neutral
palm tree	15	miscellaneous	neutral
umbrella with rain drops	15	miscellaneous	neutral
deer	14	animal	neutral
expressionless face	14	shock	negative
fog	14	miscellaneous	neutral
green salad	14	miscellaneous	neutral
hundred points	14	miscellaneous	positive
person raising hand	14	miscellaneous	neutral
red apple	14	miscellaneous	neutral
backhand index pointing left	13	miscellaneous	neutral
camera with flash	13	miscellaneous	neutral
cherries	13	miscellaneous	neutral
dizzy	13	miscellaneous	neutral
face with steam from nose	13	anger	negative
leopard	13	animal	neutral
raised hand	13	miscellaneous	neutral
shooting star	13	miscellaneous	neutral
south africa	13	geographic region	neutral
baby angel	12	miscellaneous	neutral
confetti ball	12	miscellaneous	positive
dizzy face	12	shock	negative
globe showing europe-africa	12	miscellaneous	neutral
index pointing up	12	miscellaneous	neutral
smirking face	12	happy	positive
butterfly	11	animal	neutral
cloud with rain	11	miscellaneous	negative
fireworks	11	miscellaneous	neutral
full moon with face	11	miscellaneous	neutral
gem stone	11	miscellaneous	neutral
memo	11	miscellaneous	neutral
sunrise over mountains	11	miscellaneous	neutral
upside-down face	11	anger	negative
blowfish	10	animal	neutral
candy	10	miscellaneous	neutral
chocolate bar	10	miscellaneous	neutral
face without mouth	10	anger	negative

fleur-de-lis	10	miscellaneous	neutral
frowning face with open mouth	10	anger	negative
honeybee	10	animal	neutral
owl	10	animal	neutral
paw prints	10	animal	neutral
persevering face	10	anger	negative
person swimming	10	miscellaneous	neutral
sheaf of rice	10	miscellaneous	neutral
turtle	10	animal	neutral
wrapped gift	10	miscellaneous	neutral
angry face	9	anger	negative
argentina	9	geographic region	neutral
baby	9	miscellaneous	neutral
birthday cake	9	miscellaneous	neutral
ogre	9	anger	negative
prohibited	9	miscellaneous	negative
soccer ball	9	miscellaneous	neutral
sports medal	9	miscellaneous	neutral
cityscape	8	miscellaneous	neutral
drooling face	8	happy	positive
globe showing americas	8	miscellaneous	neutral
person bowing	8	miscellaneous	neutral
person gesturing no	8	anger	negative
smiling cat face with open mouth	8	happy	positive
snake	8	animal	neutral
snowflake	8	miscellaneous	neutral
steaming bowl	8	miscellaneous	neutral
stop sign	8	miscellaneous	negative
sunrise	8	miscellaneous	neutral
writing hand	8	miscellaneous	neutral
airplane	7	miscellaneous	neutral
bird	7	animal	neutral
candle	7	miscellaneous	neutral
fish	7	animal	neutral
hear-no-evil monkey	7	miscellaneous	negative
high voltage	7	miscellaneous	neutral
last quarter moon with face	7	miscellaneous	neutral
monkey face	7	animal	neutral
neutral face	7	shock	negative
new moon face	7	miscellaneous	neutral

nose	7	miscellaneous	neutral
pistol	7	miscellaneous	neutral
post office	7	miscellaneous	neutral
raised hand with fingers splayed	7	miscellaneous	neutral
shamrock	7	miscellaneous	neutral
speaking head	7	miscellaneous	neutral
tiger face	7	animal	neutral
tornado	7	miscellaneous	neutral
tropical drink	7	miscellaneous	neutral
woman dancing	7	miscellaneous	positive
worried face	7	sad	negative
beach with umbrella	6	miscellaneous	neutral
cactus	6	miscellaneous	neutral
chequered flag	6	miscellaneous	neutral
disappointed but relieved face	6	sad	negative
dog	6	animal	neutral
eight-spoked asterisk	6	miscellaneous	neutral
house with garden	6	miscellaneous	neutral
loudspeaker	6	miscellaneous	neutral
mushroom	6	miscellaneous	neutral
pizza	6	miscellaneous	neutral
pouting face	6	anger	negative
teacup without handle	6	miscellaneous	neutral
tired face	6	anger	negative
tropical fish	6	miscellaneous	neutral
bell	5	miscellaneous	neutral
brazil	5	geographic region	neutral
bright button	5	miscellaneous	neutral
burrito	5	miscellaneous	neutral
cookie	5	miscellaneous	neutral
cooking	5	miscellaneous	neutral
cool button	5	miscellaneous	neutral
dango	5	miscellaneous	neutral
dog face	5	animal	neutral
double exclamation mark	5	shock	neutral
eight-pointed star	5	miscellaneous	neutral
french fries	5	miscellaneous	neutral
goat	5	animal	neutral
heavy check mark	5	miscellaneous	neutral
lipstick	5	miscellaneous	neutral

locked with key	5	miscellaneous	neutral
mobile phone	5	miscellaneous	neutral
mobile phone off	5	miscellaneous	neutral
mosque	5	geographic region	neutral
nerd face	5	happy	positive
person tipping hand	5	happy	positive
shaved ice	5	miscellaneous	neutral
soft ice cream	5	miscellaneous	neutral
sport utility vehicle	5	miscellaneous	neutral
strawberry	5	miscellaneous	neutral
sweat droplets	5	miscellaneous	neutral
unicorn face	5	animal	neutral
weary cat face	5	sad	negative
woman	5	miscellaneous	neutral
books	4	miscellaneous	neutral
cloud with snow	4	miscellaneous	neutral
collision	4	miscellaneous	neutral
cooked rice	4	miscellaneous	neutral
droplet	4	miscellaneous	neutral
face with head-bandage	4	sad	negative
fork and knife	4	miscellaneous	neutral
fork and knife with plate	4	miscellaneous	neutral
headphone	4	miscellaneous	neutral
hourglass with flowing sand	4	miscellaneous	neutral
ice cream	4	miscellaneous	neutral
lemon	4	miscellaneous	neutral
lollipop	4	miscellaneous	neutral
nail polish	4	miscellaneous	neutral
o button (blood type)	4	miscellaneous	neutral
pill	4	miscellaneous	neutral
potato	4	miscellaneous	neutral
shallow pan of food	4	miscellaneous	neutral
shortcake	4	miscellaneous	neutral
snowman without snow	4	miscellaneous	neutral
stuffed flatbread	4	miscellaneous	neutral
sun behind cloud	4	miscellaneous	negative
tiger	4	animal	neutral
two women holding hands	4	miscellaneous	neutral
watermelon	4	miscellaneous	neutral

afghanistan	3	geographic region	neutral
artist palette	3	miscellaneous	neutral
bento box	3	miscellaneous	neutral
bomb	3	miscellaneous	neutral
call me hand	3	miscellaneous	neutral
cheese wedge	3	miscellaneous	neutral
cityscape at dusk	3	miscellaneous	neutral
curry rice	3	miscellaneous	neutral
dashing away	3	miscellaneous	neutral
desert island	3	miscellaneous	neutral
doughnut	3	miscellaneous	neutral
face with cold sweat	3	sad	neutral
face with medical mask	3	sad	negative
fallen leaf	3	miscellaneous	neutral
family	3	miscellaneous	neutral
frog face	3	animal	neutral
front-facing baby chick	3	miscellaneous	neutral
frowning face	3	anger	negative
hamburger	3	miscellaneous	neutral
handshake	3	miscellaneous	neutral
horse racing	3	animal	neutral
hot pepper	3	miscellaneous	neutral
kuwait	3	geographic region	neutral
light bulb	3	miscellaneous	neutral
man and woman holding hands	3	miscellaneous	neutral
meat on bone	3	miscellaneous	neutral
microphone	3	miscellaneous	neutral
money-mouth face	3	miscellaneous	neutral
no mobile phones	3	miscellaneous	neutral
oden	3	miscellaneous	neutral
pencil	3	miscellaneous	neutral
person bouncing ball	3	miscellaneous	neutral
person playing handball	3	miscellaneous	neutral
popcorn	3	miscellaneous	neutral
pot of food	3	miscellaneous	neutral
poultry leg	3	miscellaneous	neutral
princess	3	miscellaneous	neutral
ribbon	3	miscellaneous	neutral
seedling	3	miscellaneous	neutral

sleepy face	3	sad	negative
slightly frowning face	3	anger	negative
slot machine	3	miscellaneous	neutral
spaghetti	3	miscellaneous	neutral
sun behind small cloud	3	miscellaneous	neutral
taco	3	miscellaneous	neutral
top hat	3	miscellaneous	neutral
two men holding hands	3	miscellaneous	neutral
violin	3	miscellaneous	neutral
ant	2	animal	neutral
automobile	2	miscellaneous	neutral
avocado	2	miscellaneous	neutral
banana	2	miscellaneous	neutral
bed	2	miscellaneous	neutral
black circle	2	miscellaneous	neutral
boxing glove	2	miscellaneous	neutral
bread	2	miscellaneous	neutral
chart increasing	2	miscellaneous	neutral
cloud with lightning and rain	2	miscellaneous	neutral
colombia	2	geographic region	neutral
comet	2	miscellaneous	neutral
cowboy hat face	2	happy	positive
croissant	2	miscellaneous	neutral
crossed fingers	2	miscellaneous	neutral
custard	2	miscellaneous	neutral
door	2	miscellaneous	neutral
drum	2	miscellaneous	neutral
exclamation question mark	2	miscellaneous	neutral
eye	2	miscellaneous	neutral
fried shrimp	2	miscellaneous	neutral
grapes	2	miscellaneous	neutral
hammer	2	miscellaneous	neutral
hot dog	2	miscellaneous	neutral
house	2	miscellaneous	neutral
india	2	geographic region	neutral
italy	2	geographic region	neutral
japan	2	geographic region	neutral

kaaba	2	geographic region	neutral
kissing cat face with closed eyes	2	love	positive
laptop computer	2	miscellaneous	neutral
left-pointing magnifying glass	2	miscellaneous	neutral
man swimming	2	miscellaneous	neutral
middle finger	2	miscellaneous	negative
musical keyboard	2	miscellaneous	neutral
national park	2	miscellaneous	neutral
no entry	2	miscellaneous	negative
page with curl	2	miscellaneous	neutral
pancakes	2	miscellaneous	neutral
pear	2	miscellaneous	neutral
people with bunny ears partying	2	miscellaneous	positive
prince	2	miscellaneous	neutral
radio button	2	miscellaneous	neutral
ring	2	miscellaneous	neutral
rooster	2	miscellaneous	neutral
santa claus	2	miscellaneous	neutral
saudi arabia	2	geographic region	neutral
small airplane	2	miscellaneous	neutral
small blue diamond	2	miscellaneous	neutral
smiling face with horns	2	anger	negative
sneezing face	2	anger	negative
sun behind large cloud	2	miscellaneous	neutral
tangerine	2	miscellaneous	neutral
television	2	miscellaneous	neutral
thumbs down	2	miscellaneous	negative
tunisia	2	geographic region	neutral
turkey	2	geographic region	neutral
two-hump camel	2	animal	neutral
volleyball	2	miscellaneous	neutral
vulcan salute	2	miscellaneous	neutral
waning crescent moon	2	miscellaneous	neutral
watch	2	miscellaneous	neutral
white heavy check mark	2	miscellaneous	neutral
wolf face	2	animal	neutral
woman facepalming	2	miscellaneous	negative

zipper-mouth face	2	miscellaneous	negative
1st place medal	1	miscellaneous	positive
airplane departure	1	miscellaneous	neutral
alien	1	miscellaneous	neutral
anchor	1	miscellaneous	neutral
anguilla	1	geographic region	neutral
anticlockwise arrows button	1	miscellaneous	neutral
back arrow	1	miscellaneous	neutral
baguette bread	1	miscellaneous	neutral
basketball	1	miscellaneous	neutral
bear face	1	animal	neutral
bikini	1	miscellaneous	neutral
blond-haired woman	1	miscellaneous	neutral
bow and arrow	1	miscellaneous	neutral
bride with veil	1	miscellaneous	neutral
bug	1	miscellaneous	neutral
bus	1	miscellaneous	neutral
bust in silhouette	1	miscellaneous	neutral
busts in silhouette	1	miscellaneous	neutral
carousel horse	1	miscellaneous	neutral
carrot	1	miscellaneous	neutral
cat	1	animal	neutral
chestnut	1	miscellaneous	neutral
chipmunk	1	animal	neutral
church	1	miscellaneous	neutral
clapper board	1	miscellaneous	neutral
classical building	1	miscellaneous	neutral
cloud with lightning	1	miscellaneous	neutral
clown face	1	happy	positive
cocktail glass	1	miscellaneous	neutral
construction	1	miscellaneous	neutral
cow	1	animal	neutral
cow face	1	animal	neutral
cross mark button	1	miscellaneous	negative
cyclone	1	miscellaneous	neutral
desert	1	miscellaneous	neutral
detective	1	miscellaneous	neutral
diamond with a dot	1	miscellaneous	neutral
direct hit	1	miscellaneous	neutral
dolphin	1	animal	neutral

double curly loop	1	miscellaneous	neutral
dragon	1	animal	neutral
ear	1	miscellaneous	neutral
ear of corn	1	miscellaneous	neutral
eggplant	1	miscellaneous	neutral
electric plug	1	miscellaneous	neutral
envelope	1	miscellaneous	neutral
european union	1	geographic region	neutral
face with thermometer	1	sad	neutral
film projector	1	miscellaneous	neutral
flashlight	1	miscellaneous	neutral
foggy	1	miscellaneous	neutral
footprints	1	miscellaneous	neutral
fountain pen	1	miscellaneous	neutral
france	1	geographic region	neutral
georgia	1	geographic region	neutral
germany	1	geographic region	neutral
glass of milk	1	miscellaneous	neutral
goal net	1	miscellaneous	neutral
gorilla	1	animal	neutral
greece	1	geographic region	neutral
green apple	1	miscellaneous	neutral
guard	1	miscellaneous	neutral
hammer and pick	1	miscellaneous	neutral
hamster face	1	animal	neutral
hatching chick	1	animal	neutral
helicopter	1	miscellaneous	neutral
high-heeled shoe	1	miscellaneous	neutral
high-speed train with bullet nose	1	miscellaneous	neutral
horse face	1	animal	neutral
hotel	1	miscellaneous	neutral
hourglass	1	miscellaneous	neutral
inbox tray	1	miscellaneous	neutral
jordan	1	geographic region	neutral
left-facing fist	1	miscellaneous	neutral

left speech bubble	1	miscellaneous	neutral
libya	1	geographic region	neutral
linked paperclips	1	miscellaneous	neutral
man playing handball	1	miscellaneous	neutral
man running	1	miscellaneous	neutral
mobile phone with arrow	1	miscellaneous	neutral
money bag	1	miscellaneous	neutral
mountain	1	miscellaneous	neutral
nauseated face	1	anger	negative
no one under eighteen	1	miscellaneous	neutral
oman	1	geographic region	neutral
oncoming bus	1	miscellaneous	neutral
outbox tray	1	miscellaneous	neutral
ox	1	animal	neutral
p button	1	miscellaneous	neutral
peace symbol	1	miscellaneous	neutral
peach	1	miscellaneous	neutral
people wrestling	1	miscellaneous	neutral
person frowning	1	anger	negative
person in bed	1	miscellaneous	neutral
person juggling	1	miscellaneous	neutral
person lifting weights	1	miscellaneous	neutral
person playing water polo	1	miscellaneous	neutral
person pouting	1	anger	negative
pile of poo	1	miscellaneous	negative
pineapple	1	miscellaneous	neutral
police car light	1	miscellaneous	neutral
poodle	1	miscellaneous	neutral
postbox	1	miscellaneous	neutral
question mark	1	miscellaneous	neutral
rabbit face	1	animal	neutral
radio	1	miscellaneous	neutral
raised back of hand	1	miscellaneous	neutral
raised fist	1	miscellaneous	neutral
red triangle pointed up	1	miscellaneous	neutral
roasted sweet potato	1	miscellaneous	neutral
rosette	1	miscellaneous	neutral
rugby football	1	miscellaneous	neutral
sake	1	miscellaneous	neutral

saxophone	1	miscellaneous	neutral
scissors	1	miscellaneous	neutral
scorpius	1	miscellaneous	neutral
scroll	1	miscellaneous	neutral
seat	1	miscellaneous	neutral
selfie	1	miscellaneous	neutral
shield	1	miscellaneous	neutral
shopping bags	1	miscellaneous	neutral
shower	1	miscellaneous	neutral
snail	1	miscellaneous	neutral
snowboarder	1	miscellaneous	neutral
sos button	1	miscellaneous	neutral
speaker high volume	1	miscellaneous	neutral
speaker low volume	1	miscellaneous	neutral
spider	1	miscellaneous	neutral
star and crescent	1	miscellaneous	neutral
studio microphone	1	miscellaneous	neutral
sushi	1	miscellaneous	neutral
syringe	1	miscellaneous	neutral
thailand	1	geographic region	neutral
three-thirty	1	miscellaneous	neutral
timer clock	1	miscellaneous	neutral
tokyo tower	1	miscellaneous	neutral
tomato	1	miscellaneous	neutral
tongue	1	miscellaneous	neutral
umbrella	1	miscellaneous	neutral
united kingdom	1	geographic region	neutral
video camera	1	miscellaneous	neutral
virgo	1	miscellaneous	neutral
white circle	1	miscellaneous	neutral
white flower	1	miscellaneous	neutral
woman biking	1	miscellaneous	neutral
woman lifting weights	1	miscellaneous	neutral
woman playing handball	1	miscellaneous	neutral
woman swimming	1	m	neutral
women wrestling	1	m	neutral

Table S2. Relationships between social media platform posts and variables of interests and excluding a highly active individual on social media. Values marked with an asterisk indicate n<5.

	Instagram®		Non-Instagram®		Total	p value
	n	%	n	%	n	
**Region**						<0.001
**UAE**	51	66.2	12	27.3	63	
**Non-UAE**	26	33.8	32	72.7	58	
**Gender***						0.012
**Male**	55	71.4	40	90.9	95	
**Female**	22	28.6	4	9.1	26	
**Species***						0.010
**Mammals**	26	33.8	4	9.1	30	
**Primates**	2	2.6	1	2.3	3	
**Birds**	49	63.6	39	88.6	88	

Table S3. Relationship of exotic animal species posts and variables of interest. Asterisked variables show that n<5.

	Species								Total	p value
	Mammals		Reptiles		Primates		Birds			
	n	%	n	%	n	%	n	%	n	
**Region***										<0.001
**UAE**	28	93.3	-	-	3	100.0	32	36.4	63	
**Non-UAE**	2	6.7	-	-	-	-	56	63.6	58	
**Gender***										<0.001
**Male**	17	56.7	-	-	1	33.3	77	87.5	95	
**Female**	13	43.3	-	-	2	66.7	11	12.5	26	
**Animal age***										<0.001
**Juvenile**	6	20.0	-	-	-	-	-	-	6	
**Adult**	24	80.0	-	-	3	100.0	88	100.0	115	
**Media form***										0.008
**Image**	28	93.3	-	-	1	33.3	78	88.6	107	
**Video**	2	6.7	-	-	2	66.7	10	11.4	14	

Table S4. Relationship between animal age and region. Region is asterisked to indicate than n<5.

	**Juvenile**		**Adult**		**Total**	**p value**
	n	%	n	%	n	
**Region***						0.016
**UAE**	6	100.0	57	49.6	63	
**Non-UAE**	-	-	58	50.4	58	
